# Association of lipoprotein‐associated phospholipase A2 mass with asymptomatic cerebral artery stenosis

**DOI:** 10.1111/jcmm.13521

**Published:** 2018-02-09

**Authors:** Youxin Wang, Bin Zhou, Pingan Zhou, Yan Yao, Qinghua Cui, Yingping Liu, Jichun Yang, Shouling Wu, Xingquan Zhao, Yong Zhou

**Affiliations:** ^1^ Beijing Key Laboratory of Clinical Epidemiology School of Public Health Capital Medical University Beijing China; ^2^ Department of Neurosurgery Baotou Eighth Hospital Baotou China; ^3^ Department of Ultrasound Yan'an University Affiliated Hospital Yan'an China; ^4^ Department of Cardiology Beijing Anzhen Hospital Capital Medical University Beijing China; ^5^ Department of Biomedical Informatics Center for Noncoding RNA Medicine MOE Key Laboratory of Cardiovascular Sciences School of Basic Medical Sciences Peking University Beijing China; ^6^ Beijing Obstetrics and Gynecology Hospital Capital Medical University Beijing China; ^7^ Pathophysiology School of Basic Medical Sciences Key Laboratory of Molecular Cardiovascular Science of the Ministry of Education Center for Non‐coding RNA Medicine Peking University Health Science Center Beijing China; ^8^ Department of Cardiology Kailuan Hospital North China University of Science and Technology Tangshan China; ^9^ Department of Neurology Beijing Tiantan Hospital Capital Medical University Beijing China; ^10^ China National Clinical Research Center for Neurological Diseases Beijing China; ^11^ Center of Stroke Beijing Institute for Brain Disorders Beijing China; ^12^ Beijing Municipal Key Laboratory of Translational Medicine for Cerebrovascular Disease Beijing China; ^13^ Beijing Institute of Heart, Lung and Blood Vessel Diseases Beijing Anzhen Hospital Capital Medical University Beijing China

**Keywords:** asymptomatic cerebral artery stenosis, cross‐sectional study, lipoprotein‐associated phospholipase A2 (Lp‐PLA2), the Asymptomatic Polyvascular Abnormalities in Community

## Abstract

Cerebral artery stenosis (CAS) is the most important causes of ischaemic stroke. Lipoprotein‐associated phospholipase A2 (Lp‐PLA2) plays 2 diverse roles in atherosclerosis (pro‐inflammatory and anti‐inflammatory), and the association between Lp‐PLA2 mass and cardiovascular or cerebrovascular events is inconsistent among previous studies. A cross‐sectional study including 2012 North Chinese adults aged ≥40 years was performed in 2010‐2011 to investigate whether Lp‐PLA2 mass is associated with asymptomatic cerebral artery stenosis (ACAS). Serum Lp‐PLA2 mass was determined by enzyme‐linked immunosorbent assay (ELISA). All participants underwent transcranial Doppler (TCD) and bilateral carotid duplex ultrasound to evaluate intracranial artery stenosis (ICAS) and extracranial arterial stenosis (ECAS). The median serum Lp‐PLA2 mass of the participants was 140.74 ng/mL (interquartile range: 131.79‐158.07 ng/mL). The adjusted odds ratio (OR) when comparing the 4th quartile to the 1st quartile of Lp‐PLA2 was 1.98 (95% confidence interval (CI): 1.42‐2.78), 1.79 (95% CI: 1.08‐2.94) and 1.87 (95% CI: 1.28‐2.73) for the occurrence of ACAS, asymptomatic ECAS and asymptomatic ICAS, respectively, after controlling for vascular risk factors. These independently significant associations remained statistically significant in the male or elderly subgroups, but not in females or middle‐aged participants. Lp‐PLA2 mass is positively correlated with subclinical atherosclerosis determined by ACAS, ICAS and ECAS in North Chinese, particularly in male and older participants, suggesting that serum Lp‐PLA2 mass might be potential biomarker for the detection of ACAS in the adults.

## 1 INTRODUCTION

Cerebral artery stenosis (CAS) is the most common pathogen of ischaemic stroke worldwide.^1^ Without treatment, cerebral artery stenosis can dramatically increase the risk of transient ischaemic attacks (TIAs), ministrokes or strokes. In China, stroke is responsible for approximately 20% of all causes of death.^2^ The prevalence of intracranial atherosclerotic disease in China is higher than that in other populations, being responsible of as far as one‐third of strokes.^3^ Currently, the diagnosis of ischaemic stroke (IS) is mainly based on the experience of clinicians and brain imaging results. However, many patients with a suspected stroke are not assessed in a timely manner (within the first few hours after the event). Thus, establishing an accurate and quick screen procedure in patients with suspected acute ischaemic stroke is very important.^4^ Identification of serum biomarkers is one of the primary strategies used to identify populations at risk of ischaemic stroke. Among these strategies, lipoprotein‐associated phospholipase A2 (Lp‐PLA2) has been identified as a promising soluble blood‐based biomarker.^5^

Lp‐PLA2 is a type of serine lipase, majority of which bound to low‐density lipoproteins (LDL) and less of which bound to high‐density lipoprotein (HDL) or very low‐density lipoprotein (VLDL).^6^ Paradoxically, the role of Lp‐PLA2 in the progression of atherosclerosis is controversial.^7^, ^8^, ^9^ As a well‐established pro‐inflammatory factor, Lp‐PLA2 is extensively involved in the progression of atherosclerosis, including plaque formation, development and rupture.^10^ A high level of Lp‐PLA2 has been proved to be a positive risk factor for cardiovascular and cerebrovascular events in multiple large‐scale population studies.^4^, ^11^, ^12^, ^13^, ^14^, ^15^ The Northern Manhattan Study has reported a correlation between Lp‐PLA2 mass and increased risk of first atherosclerotic stroke among non‐Hispanic White participants during an 11‐year follow‐up (adjusted hazard ratio [HR], 1.55; 95% CI, 1.17‐2.04).^4^ In a pooling analysis of 32 prospective studies, Lp‐PLA2 mass was found to be associated with risk of coronary heart disease and vascular death, with adjusted relative risk (RR) of 1.11 (1.07‐1.16) for coronary heart disease, 1.14 (1.02‐1.27) for ischaemic stroke, 1.13 (1.05‐1.22) for vascular mortality and 1.10 (1.03‐1.18) for non‐vascular mortality.^11^ In contrast, some studies demonstrated a decrease in Lp‐PLA2 enzymatic activity in patients with acute myocardial infarction and no correlation with premature coronary atherosclerosis;^16^, ^17^ therefore, the underlying mechanism remains unclear.

Lp‐PLA2 is well recognized as a potential biomarker for the vascular inflammation and formation of rupture‐prone plaques.^18^ Lp‐PLA2 is considered to predict the risk of not only first‐ever but also recurrent strokes,^19^, ^20^, ^21^, ^22^ but it does not correlate with acute cerebrovascular ischaemic diseases.^23^ This discrepancy might be due to the heterozygous phenotypes of stroke and the paradoxical roles of Lp‐PLA2 in the inflammation. Lp‐PLA2 mass is significantly associated with isolated ICAS and concurrent extra‐intracranial stenosis but not related to isolated ECAS, in a stroke‐free hypertension population from North China.^24^ In this study, we examined asymptomatic cerebral artery stenosis (ACAS), an intermediate indicator that can remain silent for a long period before the occurrence of ischaemic stroke,^25^ and suggested that aberrant increase in Lp‐PLA2 mass might be associated with ACAS in a vascular disease‐free population. The objective of this study was to investigate the association between serum levels of Lp‐PLA2 with ACAS in a Chinese cohort, with the aim of identifying useful and novel biomarkers for the prediction of stroke at an early stage.

## 2 METHODS

### 2.1 Study design and population

The participants and design of the Asymptomatic Polyvascular Abnormalities in Community (APAC) study can be referred to your previously description.^26^ The APAC study included a random sample of 5440 participants aged ≥40 years. The participants were selected from the baseline population from the Kailuan Study and included 101 510 current employees and retirees from the Kailuan (Group) Co. Ltd.^26^ The APAC study protocol has been previously reported in detail.^26^ Among the 5440 participants, 2012 with complete demographic and blood sample information were randomly selected to investigate the association between Lp‐PLA2 mass and ACAS.

### 2.2 Measurement of Lp‐PLA2

The correlation between Lp‐PLA2 mass and Lp‐PLA2 activity was about 0.50.^11^ In addition, the Lp‐PLA2 mass and Lp‐PLA2 activity had similar predictive power for the cardiac death^27^ and stroke.^28^ Therefore, we measured Lp‐PLA2 mass only.

Venous bloods were collected in the fasting condition, and EDTA was used as an anticoagulant. The blood samples were centrifuged for 5 minutes at 500 × g. within 2 hours of field collection, and serum was separated and placed in microcentrifuge tubes. Blood samples had unified number for each participant and were stored at −80°C. To reduce the interassay error and measurement error, the Lp‐PLA2 mass for all participants was assessed simultaneously by a professional technician using the human Lp‐PLA2 enzyme immunoassay kit (CUSABIO, Wuhan, China) at Beijing Tiantan Hospital, Capital Medical University, Beijing, China, according to the manufacturer's instructions.

### 2.3 Assessment of cerebral artery stenosis

Intracranial artery stenosis (ICAS) was assessed through the use of a transcranial Doppler (TCD) by 2 independent experienced neurologists using portable machines (EME, Companion, Nicolet), according to standardized protocol and diagnosis criteria.^26^ Artery stenosis was defined by the peak systolic flow velocity as follows: >140 cm per second for the middle cerebral artery, or >120 cm per second for the anterior cerebral artery, or >100 cm per second for the posterior cerebral artery and vertebral‐basilar artery or >120 cm per second for the ICAS. Except for velocity criteria, the presence of turbulence or background noise, and whether the abnormal velocity was considered to be segmental.

Every participant also underwent a bilateral carotid duplex ultrasound (Philips iU‐22 ultrasound system, Philips Medical Systems, Bothell, WA, USA) to assess extracranial arterial stenosis (ECAS). Bilateral ECAS arteries included common carotid arteries, carotid bifurcation, the internal carotid artery and the external carotid artery. All participants were examined in the supine position with the head turned to the contralateral side. Both sides of the carotid arteries were evaluated for the presence of ECAS (≥50%), which was graded based on recommendations from the Society of Radiologists in Ultrasound Consensus Conference.^29^ ACAS was defined by the presence of at least one of ECAS or ICAS.

### 2.4 Covariates

Demographic variables including age, sex and history of hypertension, diabetes mellitus and dyslipidaemia were collected via questionnaires. All participants were divided into 2 groups based on their ages: 40‐59 years and ≥60 years. Information regarding disease history, including hypertension, diabetes mellitus or hyperlipidaemia, as well as smoking history, which was classified as “yes” or “no” was also collected via questionnaires. Weights (accurate to 0.1 kg) and heights (accurate to 0.1 cm) were measured during the physical examination, and body mass index (BMI) values were calculated. Hypertension was defined as the presence of hypertension history, use of antihypertensive medication, a systolic blood pressure (SBP) ≥140 mm Hg or a diastolic blood pressure (DBP) ≥90 mm Hg. Diabetes mellitus was defined by self‐reported history, current use of insulin or oral hypoglycaemic agents, or a fasting blood glucose level ≥7.0 mmol/L (126 mg/dL). Dyslipidaemia was defined as a self‐reported history, current use of cholesterol‐lowering medication, total cholesterol (TC) level ≥220 mg/dL or triglycerides (TG) ≥150 mg/dL or low‐density lipoprotein cholesterol (LDL‐C) ≥160 mg/dL. C‐Reactive protein (CRP) was measured by high‐sensitivity nephelometry assay (Cias Latex CRP‐H; Kanto Chemical Co. Inc, Tokyo, Japan). CRP concentrations were categorized into 2 groups according to the guideline.^30^ All blood examinations were performed at the central laboratory of Kailuan Hospital.

### 2.5 Statistical analyses

Continuous variables are presented as the mean ± standard deviation or median together with interquartile range, whereas categorical variables are presented number together with percentages. Continuous variables were compared using analysis of variance (anova) for normally distributed variables and nonparametric approach for skewed distributed variables. The intergroup differences in categorical variables were compared using chi‐square tests. We used logistic regression to determine the association between the Lp‐PLA2 mass and cerebral artery stenosis risk and represented as odds ratio (OR) and 95% confidence interval (CI). Known confounding factors including age, sex, BMI, current smoker, alcohol use, hypertension, diabetes mellitus and dyslipidaemia were controlled in the logistic regression. All statistical tests were 2‐sided, and *P* < .05 was considered to be statistically significant. All analyses were performed with SAS (version 9.1; SAS Institute, Cary, NC, USA) software.

### 2.6 Ethics statement

This study was approved by the Ethics Committees of Beijing Tiantan Hospital, Capital Medical University. All participants signed informed consent forms before the participation in this study.

## 3 RESULTS

This study consisted of 1482 males and 530 females with a mean age of 60.5 ± 11.7 years. The median Lp‐PLA2 mass was 141.74 ng/mL (interquartile range, 131.79‐158.07 ng/mL), which divided to 4 quartiles, 1st quartile (<131.79 ng/mL), 2nd quartile (131.79‐140.74 ng/mL), 3rd quartile (140.75‐158.07) and 4th quartile (>158.07 ng/mL). ACAS was detected in 479 (23.6%) participants (363 were ICAS and 191 were ECAS).

We summarized the baseline characteristics of all participants in Table 1. In this stroke‐free cohort, participants with ACAS were elder (60.45 vs 59.16 years) and included more males than females (79.75% vs 20.25%). Moreover, participants in the ACAS group were more likely than the non‐ACAS group to suffer from hypertension and diabetes mellitus. The biochemical indicators of Lp‐PLA2, glucose (GLU), total cholesterol (CHOL), C‐reactive protein (CRP) and homocysteine (HCY) in the ACAS group were significant higher than those in the non‐ACAS group (all *P* < .05).

**Table 1:** Baseline characteristics of ICAS, ECAS and ACAS groups

	Non‐ACAS (n = 1539)	ACAS (n = 473)	ICAS (n = 358)	ECAS (n = 186)
Sociodemographic factors				
Male sex, n (%)	1105 (71.80)	377 (79.70)^b^	270 (75.42)	170 (91.40)^b^
Age (Y)	57.41 (51.18, 66.45)	63.12 (54.14, 74.78)^b^	63.89 (55.04, 74.25)^b^	69.72 (54.25, 78.71)^b^
Age category, n (%)				
<60 y	918 (82.55)	194 (14.45)^b^	140 (12.59)^b^	65 (5.85)^b^
≥60 y	621 (69.00)	279 (31.00)^b^	218 (24.22)^b^	121 (13.44)^b^
Vascular risk factors				
Hypertension, n (%)	821 (53.35)	342 (72.30)^b^	279 (77.93)^b^	120 (64.52)
Diabetes mellitus, n (%)	223 (14.49)	111 (23.47)^b^	94 (26.26)^b^	42 (22.58)^a^
Dyslipidaemia, n (%)	811 (52.70)	257 (54.33)	204 (56.98)	93 (50.00)
Body mass index (kg/m^2^)	24.77 (22.72, 26.89)	24.86 (22.85, 27.34)	25.00 (23.11, 27.34)	24.42 (22.15, 27.08)
Smoke, n (%)	575 (37.36)	192 (40.59)	133 (37.15)	89 (47.85)^b^
Alcohol, n (%)	256 (16.63)	68 (14.38)	49 (17.79)	27 (14.52)
Exercise, n (%)	612 (39.77)	202 (42.71)	161 (44.97)	82 (44.09)
Laboratory measurements				
Lp‐PLA2 mass (ng/mL)	139.35 (131.19, 155.00)	146.10 (135.18, 169.83)^b^	145.91 (135.45, 171.86)^b^	147.98 (135.06, 177.69)^b^
Lp‐PLA2 mass category, n (%)				
<200 ng/mL	1403 (77.51)	407 (22.49)^b^	306 (16.91)^b^	158 (8.73)^b^
≥200 and <223 ng/mL	40 (70.18)	17 (29.82)^b^	12 (21.05)^b^	8 (14.04)^b^
≥223 ng/mL	96 (66.21)	49 (33.79)^b^	40 (27.59)^b^	20 (13.79)^b^
GLU (mmol/L)	5.24 (4.85, 5.83)	5.42 (4.90, 6.50)^b^	5.50 (4.95, 6.61)^b^	5.40 (4.80, 6.43)
CHOL (mmol/L)	5.06 (4.43, 5.78)	5.19 (4.58, 5.86)^a^	5.23 (4.64, 5.97)^b^	5.10 (4.53, 5.10)
TG (mmol/L)	1.30 (0.93, 1.90)	1.32 (0.94, 1.89)	1.33 (1.00, 1.96)	1.21 (0.86, 1.71)
HDL‐C (mmol/L)	1.56 (1.29, 1.91)	1.53 (1.25, 1.89)	1.53 (1.25, 1.88)	1.59 (1.30, 1.91)
LDL‐C (mmol/L)	2.60 (2.14, 3.12)	2.72 (2.19, 3.18)	2.74 (2.18, 3.19)	2.76 (2.19, 3.19)
CRP (mg/L)	1.10 (0.56, 2.30)	1.40 (0.70, 3.00)^b^	1.44 (0.80, 3.14)^b^	1.41 (0.70, 3.40)^b^
HCY (μmol/L)	14.70 (11.00, 20.70)	16.05 (11.80, 22.72)^b^	16.00 (11.60, 21.80)^a^	18.15 (13.00, 24.15)^b^
UA (μmol/L)	295.00 (237.00, 358.00)	313.00 (250.00, 366.00)	303.50 (250.95, 370.00)	309.00 (264.00, 362.00)

ICAS, intracranial artery stenosis; ECAS, extracranial artery stenosis; ACAS, asymptomatic cerebral artery stenosis.

a Denotes a significance level of *P* = .05.

b Denotes a significance level of *P* = .01.

The association between artery stenosis and Lp‐PLA2 mass was summarized in Table 2. Compared to the participants in the 1st quartile of Lp‐PLA2 mass, there was an increased risk of ACAS among participants in the 2nd quartile (OR, 1.47; 95% CI, 1.05‐2.05), 3rd quartile (OR = 1.64; 95% CI, 1.18‐2.29) and 4th quartile (OR, 1.98; 95% CI, 1.42‐2.78), after controlling for sex, age, smoking, alcohol, exercise, hypertension, hyperlipidaemia, diabetes and BMI using Lp‐PLA2 mass quartiles. The association between Lp‐PLA2 mass and ACAS was similar in the 2 stenosis subgroups. There was a trend towards an increased risk of asymptomatic ECAS in comparison with that of the 1st quartile, with the OR = 1.32 (95% CI, 0.79‐2.19), 1.42 (95% CI, 0.86‐2.35) and 1.79 (95% CI, 1.08‐2.94) in the 2nd quartile, 3rd quartile and 4th quartile, respectively, and a greater prominent increase was observed for asymptomatic ICAS in comparison with that of the 1st quartile, 2nd quartile (OR, 1.50; 95% CI, 1.02‐2.19), 3rd quartile (OR, 1.69; 95% CI, 1.15‐2.43) and 4th quartile (OR, 1.87; 95% CI, 1.28‐2.73), after controlling for age, sex, hypertension, diabetes mellitus, dyslipidaemia, smoking, alcohol and BMI. When CRP was further adjusted, these associations remain statistically significant (Table 2), indicating that the associations between Lp‐PLA2 mass and ACAS, ECAS and ICAS was independent to CRP.

**Table 2:** Association between Lp‐PLA2 mass and ACAS, ECAS or ICAS

	Model 1	Model 2	Model 3	Model 4	Model 5		Model 6	
					Male	Female	Age <60 y	Age ≥ 60 y
ACAS								
Quartile 1	1	1	1	1	1	1	1	1
Quartile 2	**1.59 (1.14‐2.20)**	**1.47 (1.06‐2.04)**	**1.47 (1.05‐2.05)**	**1.46** (**1.05‐2.05**)	1.42 (0.97‐2.08)	1.55 (0.76‐3.17)	1.37 (0.90‐2.10)	1.72 (0.99‐2.99)
Quartile 3	**2.00 (1.45‐2.75)**	**1.67 (1.20‐2.32)**	**1.64 (1.18‐2.29)**	**1.63** (**1.17‐2.28**)	**1.63 (1.11‐2.37)**	1.56 (0.78‐3.13)	**1.90 (1.23‐2.92)**	1.65 (0.97‐2.79)
Quartile 4	**2.75 (2.02‐3.75)**	**1.99 (1.43‐2.76)**	**1.98 (1.42‐2.78)**	**1.96** (**1.40‐2.75**)	**2.00 (1.36‐2.93)**	1.75 (0.86‐3.54)	1.56 (0.96‐2.55)	**2.76 (1.66‐4.57)**
ICAS								
Quartile 1	1	1	1	1	1	1	1	1
Quartile 2	**1.65 (1.14‐2.39)**	**1.52 (1.05‐2.21)**	**1.50 (1.02‐2.19)**	**1.49** (**1.02‐2.18**)	1.40 (0.90‐2.19)	1.72 (0.82‐3.63)	1.35 (0.82‐2.21)	1.80 (0.98‐3.31)
Quartile 3	**2.13 (1.49‐3.05)**	**1.73 (1.20‐2.50)**	**1.67 (1.15‐2.43)**	**1.66** (**1.14‐2.42**)	**1.65 (1.06‐2.56)**	1.60 (0.76‐3.33)	**2.04 (1.25‐3.34)**	1.60 (0.89‐2.88)
Quartile 4	**2.72 (1.92‐3.86)**	**1.88 (1.30‐2.73)**	**1.87 (1.28‐2.73)**	**1.84** (**1.25‐2.69**)	**1.77 (1.08‐2.78)**	1.92 (0.91‐4.01)	1.37 (0.76‐2.45)	**2.61 (1.50‐4.57)**
ECAS								
Quartile 1	1	1	1	1	1	1	1	1
Quartile 2	1.47 (0.89‐2.41)	1.32 (0.79‐2.19)	1.32 (0.79‐2.19)	1.31 (0.79‐2.18)	1.30 (0.77‐2.22)	1.39 (0.22‐8.58)	1.35 (0.69‐2.64)	1.49 (0.67‐3.32)
Quartile 3	**1.79 (1.10‐2.90)**	1.42 (0.86‐2.34)	1.42 (0.86‐2.35)	1.40 (0.85‐2.32)	1.37 (0.81‐2.32)	1.87 (0.34‐10.17)	1.45 (0.71‐2.97)	1.70 (0.80‐3.62)
Quartile 4	**2.74 (1.73‐4.33)**	**1.79 (1.09‐2.94)**	**1.79 (1.08‐2.94)**	**1.73** (**1.05‐2.86**)	**1.72 (1.02‐2.91)**	2.04 (0.37‐11.12)	1.68 (0.79‐3.58)	**2.48 (1.20‐5.10)**

ACAS, asymptomatic cerebral artery stenosis; ECAS, extracranial artery stenosis; ICAS, intracranial artery stenosis.

Bold OR (95% CI) indicates statistically significant.

Model 1: Unadjusted; Model 2: Adjusted for age and sex; Model 3: Adjusted for age, sex, hypertension, diabetes mellitus, dyslipidaemia, smoking, alcohol and BMI; Model 4: Adjusted for age, sex, hypertension, diabetes mellitus, dyslipidaemia, smoking, alcohol, BMI and CRP; Model 5: Adjusted for all (except sex, CRP) and stratified by age; Model 6: Adjusted for all (except age, CRP) and stratified by sex.

In the stratified analysis, compared to the participants in the 1st quartile of Lp‐PLA2 mass, increased risk of ACAS in the 4th quartile was statistically significant in the males (OR, 2.00; 95% CI, 1.36‐2.93) but not significant in females (OR, 1.75; 95% CI, 0.86‐3.54), and was significant in participants aged <60 years (OR, 2.76; 95% CI, 1.66‐4.57) but not significant in participants aged ≥60 years (OR, 1.56; 95% CI, 0.96‐2.55). For stratification analyses, the association remained statistically significant only in males and in the elder (aged ≥60 years).

## 4 DISCUSSION

The present study indicated that the serum Lp‐PLA2 mass was elevated with ACAS. The results demonstrated that elevated Lp‐PLA2 mass is independently associated with the increased risk of ACAS (either ICAS or ECAS), and the independent association is statistically significant in the male or elderly subgroups, but not significant in females or middle‐aged participants. To our knowledge, this is the first attempt to investigate the association between Lp‐PLA2 mass and the presence of subclinical atherosclerosis in a general and asymptomatic Chinese population, and the only study addressing the association between Lp‐PLA2 mass and ACAS, ICAS or ECAS in terms of general population levels.

We found that a high Lp‐PLA2 mass (eg 4th quartile) was associated with an increased risk of ACAS. Our findings were in part consistent with the findings reported in another population (stroke‐free hypertensive patients), which showed that Lp‐PLA2 mass was independently associated with isolated ICAS (OR, 2.30; 95% CI, 1.14‐4.64) and concurrent extra‐intracranial stenosis (OR, 3.93; 95% CI, 1.62‐9.51) but not isolated ECAS (OR, 1.54; 95% CI, 0.68‐3.48).^24^ Recently, a systematic review was performed to explore the association between Lp‐PLA2 and markers of subclinical cardiovascular disease; the results showed that 3 of 6 studies favour the relationship between Lp‐PLA2 and coronary artery calcification (CAC), 3 of 5 studies favour the relationship between Lp‐PLA2 and carotid intima‐media thickness (CIMT), and one of 2 studies favour the association between Lp‐PLA2 and endothelial dysfunction.^31^ Taken together, these findings indicate a variable association of Lp‐PLA2 subclinical atherosclerosis. Thus, Lp‐PLA2 provides an important opportunity to appropriately classify the participants who are actually at high risk but mistakenly classified, and these findings have broad implications for future public health and clinical practice.

The present study revealed that the associations between Lp‐PLA2 mass and ACAS were statistically significant in males but not in females. This finding is consistent with the results of the Dallas Heart Study, which has shown that Lp‐PLA2 mass is modestly associated with CAC in males but not in females.^32^ Our finding is similar to the results of the Chinese Multiprovincial Cohort Study—Beijing Project, which showed that Lp‐PLA2 activity is independently associated with the development of subclinical atherosclerosis in men but not in women.^33^ Oestrogen has been reported to be associated with the low expression or activity of Lp‐PLA2;^34^ therefore, the effects of sex hormones might in part explain this sex discrepancy. Similar to other studies with the distinct gender composition (27% females in the Veterans Affairs Diabetes Trial study and 29% females in the Coronary Artery Risk Development in Young Adults study),^35^, ^36^ the small proportion of females in this study cohorts (26.3%) may suggest that insufficiency in statistical power might also contribute to the non‐significant association in females. Further larger, more robust studies are urged to clarify the association between ACAS risk and Lp‐PLA2.

In the present study, the association between ICAS or ECAS was significant only in elderly participants (aged ≥60 years) but not in middle‐aged participants (<60 years). This finding is consistent with results from a report investigating a Japanese population,^37^ but it is inconsistent with results from a study performed in Istanbul, Turkey, which showing that serum Lp‐PLA2 mass is significantly increased in participants with subclinical coronary atherosclerosis compared to control patients, and Lp‐PLA2 mass is positively correlated with the total number of plaques and diseased arteries in a young adult population (<45 years).^38^ The reason that Lp‐PLA2 mass was not significantly associated with ACAS in middle‐aged participants remains unclear; however, the lower prevalence of ACAS in middle‐aged participants (14.45%) compared to that in the elderly participants (31.00%) might in part lead to the insufficient statistical power and thus make the association non‐significant. Further in‐depth studies are needed to explain such discrepancy.

There are several limitations to the present study. Firstly, the study relies on TCD for the diagnosis of intracranial stenoses, and not on conventional angiography, which is considered the gold standard. TCD does not collect information regarding the histopathological nature of the lesions related to vessel narrowing, and patients with non‐atherosclerotic vascular pathology might have been included. Secondly, the blood samples were stored for 3‐4 years at −80°C. However, it has been reported that Lp‐PLA2 can be relatively stable over time when stored at <−70°C.^39^ Thirdly, the design of cross‐sectional study limited the power to interpret the cause‐effect association between the higher serum Lp‐PLA2 mass and ACAS. Finally, most of the participants were coal workers, thus potentially limiting the ability to generalize our results to other populations.

## 5 CONCLUSION

In conclusion, we found that Lp‐PLA2 mass was positively correlated with subclinical atherosclerosis determined by ACAS, ICAS and ECAS, particularly in male and older participants. Considering that elevation Lp‐PLA2 contributes about 2‐fold risk for strokes or coronary artery disease, testing for Lp‐PLA2 might be a supplementary evaluation tool to classical cardiovascular risk assessment.

## CONFLICT OF INTEREST

The authors declare that they have no conflict of interest.
